# Supplementary material for: Modeling the dynamics of Plasmodium falciparum gametocytes in humans during malaria infection
Source: eLife. 2019 Oct 29;8:e49058. doi: 10.7554/eLife.49058 (PMC6819085; doi:10.7554/eLife.49058)
Supplement: Transparent reporting form [file elife-49058-transrepform.pdf]

eLIFE
1st Floor
24 Hills Road
Cambridge CB2 1JP, UK
P 01223 855340
W elifesciences.org
T @elife

eLife's transparent reporting form

We encourage authors to provide detailed information *within their submission* to facilitate the interpretation and replication of experiments. Authors can upload supporting documentation to indicate the use of appropriate reporting guidelines for health-related research (see EQUATOR Network), life science research (see the BioSharing Information Resource), or the ARRIVE guidelines for reporting work involving animal research. Where applicable, authors should refer to any relevant reporting standards documents in this form.

If you have any questions, please consult our Journal Policies and/or contact us: editorial@elifesciences.org.

Sample-size estimation

- You should state whether an appropriate sample size was computed when the study was being designed
- You should state the statistical method of sample size computation and any required assumptions
- If no explicit power analysis was used, you should describe how you decided what sample (replicate) size (number) to use

Please outline where this information can be found within the submission (e.g., sections or figure legends), or explain why this information doesn't apply to your submission:

The statistical methods and the sample size of the posterior distributions for the posterior distributions presented in the main text are provided in the Materials and Methods. As this is a mathematical modelling study based on published clinical data, there are no further statistical details to report. Our analysis is designed to extract the maximum possible insight from available data.

Replicates

- You should report how often each experiment was performed
- You should include a definition of biological versus technical replication
- The data obtained should be provided and sufficient information should be provided to indicate the number of independent biological and/or technical replicates
- If you encountered any outliers, you should describe how these were handled
- Criteria for exclusion/inclusion of data should be clearly stated
- High-throughput sequence data should be uploaded before submission, with a private link for reviewers provided (these are available from both GEO and ArrayExpress)

Please outline where this information can be found within the submission (e.g., sections or figure legends), or explain why this information doesn't apply to your submission:

This is a modelling study based on published clinical data, so the questions are not directly applicable. That said, we applied our mathematical model to every patient's data as published in the original study, and have reported all results, including any occurrences of poor model fit(s) to particular individual patients.

Statistical reporting

- Statistical analysis methods should be described and justified
- Raw data should be presented in figures whenever informative to do so (typically when N per group is less than 10)
- For each experiment, you should identify the statistical tests used, exact values of N, definitions of center, methods of multiple test correction, and dispersion and precision measures (e.g., mean, median, SD, SEM, confidence intervals; and, for the major substantive results, a measure of effect size (e.g., Pearson's r, Cohen's d)
- Report exact p-values wherever possible alongside the summary statistics and 95% confidence intervals. These should be reported for all key questions and not only when the p-value is less than 0.05.

Please outline where this information can be found within the submission (e.g., sections or figure legends), or explain why this information doesn't apply to your submission:

The mathematical model and statistical methods are described in full in the Materials and Methods. The clinical data are provided in Figure 1-3 in the main text, and also as raw data in spreadsheet format with the computer code repository (publicly available at https://doi.org/10.26188/5cde4c26c8201). Statistical measures, such as median, credible interval and prediction interval, are clearly indicated wherever presented.

(For large datasets, or papers with a very large number of statistical tests, you may upload a single table file with tests, Ns, etc., with reference to sections in the manuscript.)

Group allocation

- Indicate how samples were allocated into experimental groups (in the case of clinical studies, please specify allocation to treatment method); if randomization was used, please also state if restricted randomization was applied
- Indicate if masking was used during group allocation, data collection and/or data analysis

Please outline where this information can be found within the submission (e.g., sections or figure legends), or explain why this information doesn't apply to your submission:

This is a modelling work based on published clinical data, so this information is not applicable. The group allocation information about the clinical study has been provided in Collins et al. 2018. Journal of Clinical Investigation (doi:10.1172/JCI98012).

Additional data files ("source data")

- We encourage you to upload relevant additional data files, such as numerical data that are represented as a graph in a figure, or as a summary table
- Where provided, these should be in the most useful format, and they can be uploaded as "Source data" files linked to a main figure or table
- Include model definition files including the full list of parameters used
- Include code used for data analysis (e.g., R, MatLab)
- Avoid stating that data files are "available upon request"

Please indicate the figures or tables for which source data files have been provided:

eLife Sciences Publications, Ltd is a limited liability non-profit non-stock corporation incorporated in the State of Delaware, USA, with company number 5030732, and is registered in the UK with company number FC030576 and branch number BR015634 at the address 1st Floor, 24 Hills Road, Cambridge CB2 1JP | August 2014

All numerical data and computer code (approximately 2.5GB) have been made publicly available at https://doi.org/10.26188/5cde4c26c8201. An instruction file is also provided along with the code to help readers understand the code and data and easily use them to reproduce any result in the manuscript and the supplementary information. Links to this information have been included in the Materials and Methods.
